# Efficient Privacy-Preserving Data Sharing for Fog-Assisted Vehicular Sensor Networks

**DOI:** 10.3390/s20020514

**Published:** 2020-01-16

**Authors:** Yang Ming, Xiaopeng Yu

**Affiliations:** School of Information Engineering, Chang’an University, Xi’an 710064, China; 2017124021@chd.edu.cn

**Keywords:** vehicular sensor networks, fog computing, data sharing, privacy preserving

## Abstract

Vehicular sensor networks (VSNs) have emerged as a paradigm for improving traffic safety in urban cities. However, there are still several issues with VSNs. Vehicles equipped with sensing devices usually upload large amounts of data reports to a remote cloud center for processing and analyzing, causing heavy computation and communication costs. Additionally, to choose an optimal route, it is required for vehicles to query the remote cloud center to obtain road conditions of the potential moving route, leading to an increased communication delay and leakage of location privacy. To solve these problems, this paper proposes an efficient privacy-preserving data sharing (EP${}^{2}$DS) scheme for fog-assisted vehicular sensor networks. Specifically, the proposed scheme utilizes fog computing to provide local data sharing with low latency; furthermore, it exploits a super-increasing sequence to format the sensing data of different road segments into one report, thus saving on the resources of communication and computation. In addition, using the modified oblivious transfer technology, the proposed scheme can query the road conditions of the potential moving route without disclosing the query location. Finally, an analysis of security suggests that the proposed scheme can satisfy all the requirements for security and privacy, with the evaluation results indicating that the proposed scheme leads to low costs in computation and communication.

## 1. Introduction

Vehicular sensor networks (VSNs) [1,2,3], that is, a combination of wireless communication given by vehicular ad hoc networks [4] and the sensing devices installed in the vehicle, can improve traffic conditions in urban cities, and have recently received considerable attention. In VSNs, the vehicles equipped with sensing devices can record a myriad of data reports on the road conditions and environment situations, and these data reports need be uploaded to the remote cloud center [5,6] for processing and analyzing. In addition, vehicles often need to query the road conditions of potential moving routes at remote cloud centers. However, uploading a large amount of data reports to the cloud data center consumes heavy bandwidth, and leads to an increased communication delay.

Recently, fog computing [7] has been proposed to extend the capabilities of cloud computing [8] near vehicles [9], which can locally handle the data reports uploaded by vehicles. These new properties will bring about benefits such as location awareness and low latency. Fog computing has already been used to provide low latency services in vehicular sensor networks, such as navigation services [10] and surface condition monitoring [11].

A typical architecture of fog-assisted vehicular sensor networks (F-VSNs) [12,13,14] contains the trusted authority, cloud center, fog nodes, and vehicles. The trusted authority is responsible for generating system parameters, and the registration of all entities (cloud center, fog nodes and vehicles). The cloud center provides centralized control with strong computing power and large storage capacity from a remote location. Fog nodes have available computing, storage, and communication resources [15], which is deployed at the edge of networks with physical proximity to vehicles, playing as the bridge across the vehicles and the cloud center. Vehicles are installed with a variety of smart sensors that can sense road conditions and environmental parameters. F-VSNs allows some computations and processing to be performed at the fog nodes, greatly reducing the consumption of communication time and energy.

Although F-VSNs brings a great deal of benefits and conveniences, there still exist several issues in terms of data collection and data query. Specifically, vehicles generate a large amount of sensory data reflecting the road conditions and environment situations, and need to upload the sensory data to cloud center for further processing and analyzing, which brings heavy computation and communication costs. To solve this problem, data aggregation technology, which is designed to aggregate multiple data into one report, has recently received more and more attention.

However, using the existing data aggregation schemes [16,17,18,19,20,21,22] cannot determine the number of data reports produced in each road segment, and cannot compute the average sensory data in each road segment. To solve the problem, the scheme [23] exploits the Chinese remainder theorem and Paillier cryptosystem to calculate the average sensory data in each segments; however, it brings heavy computation and communication costs. In addition, to choose an optimal route, vehicles often query about the road conditions of the potential moving routes, but the query reports uploaded by vehicles are tightly associated with the query location, and thus the query location could be disclosed.

The oblivious transfer [24,25], homomorphic encryption technology [26,27], and proxy re-encryption technique [23] have been exploited to hide the query location. However, it is worth noting that the computation and communication costs by the schemes [24,25] is directly proportional to the data dimension, the schemes [26,27] do not support the scenario with high vehicle density, and the scheme [23] needs heavy computation and communication costs.

### 1.1. Our Contributions

To solve the aforementioned problems, this paper proposes an efficient privacy-preserving data sharing (EP${}^{2}$DS) scheme for fog-assisted vehicular sensor networks. The main contributions of this paper are as follows:

- First, the proposed EP${}^{2}$DS scheme exploits the super-increasing sequence [20] for achieving multi-dimensional data aggregation, while calculating the average sensory data in each road segment, greatly saving on the resources of communication and computation.
- Secondly, by utilizing the modified oblivious transfer [28], the proposed EP${}^{2}$DS scheme is able to query about the road conditions of the potential moving routes without disclosing the query location.
- Thirdly, an analysis of security indicates that the proposed EP${}^{2}$DS scheme is proven to be secure under elliptic curve discrete logarithm (ECDL) assumption in the random oracle model and satisfies all the requirements for security and privacy.
- Finally, the performances of computation and communication in costs are evaluated through quantitative calculations, with the results that the proposed EP${}^{2}$DS scheme is of more efficiency than others.

### 1.2. Organization

This paper is organized as follows. The related work is surveyed in Section 2. We introduce the background in Section 3. The concrete scheme is proposed in Section 4. Section 5 provides an analysis of the security. In Section 6, the performance evaluation is performed. Section 7 concludes the paper.

## 2. Related Works

Some works closely related to this paper are briefly reviewed below.

In F-VSNs, massive sensory data is produced in each data dimension, and needs to be uploaded for further processing and analysis; data aggregation schemes [16,17,18,19,20,21,22,23] have received considerable attention recently, and are roughly classified into two categories: single-dimensional data aggregation [16,17,18,19] and multi-dimensional data aggregation [20,21,22,23]. Zhuo et al. [16] introduced a data aggregation scheme, which protects each involved entity’s identity privacy, and allows the requester to examine the correctness of the obtained results. Rabieh et al. [17] employed the data aggregation technique to find out the routes for the vehicle to be in each road segment; however, it only can calculate the data aggregation result, and cannot recover the content in each data dimension.

Xu et al. [18] constructed a privacy-preserving data aggregation scheme that can classify messages based on where and when the sensor data is collected, and aggregate the data collected in the same area and period. Sun et al. [19] designed a data aggregation mechanism considering data integrity and access control. However, the schemes [16,17,18,19] are unable to determine the number of the data reports produced in each data dimension, and further fail to calculate the average sensory data in each data dimension. Lin et al. [20] integrated the perturbation technique and super-increasing sequence to combine multiple aggregated data into one data report to improve the energy efficiency.

Lu et al. [21] employed the homomorphic Paillier encryption, one-way hash chain technique and Chinese remainder theorem to achieve lightweight multi-dimensional data aggregation. On the basis of the super-increasing sequence and modified homomorphic Paillier encryption, Wang et al. [22] introduced a multi-subtasks aggregation scheme, in which each aggregated datum is mapped to a specific area and period. Kong et al. [23] designed a privacy-preserving multi-dimensional data sharing scheme using the Chinese remainder theorem and modified Paillier encryption, with counting the number of the sensory data collected at each segments and calculating the average sensory data in each segment.

Although schemes [20,21,22,23] are able to calculate the average sensory data in each data dimension, they bring heavy computation costs and communication overhead. In addition, the query vehicle usually wants to know the road conditions of the potential moving route, which could lead to that the query location being disclosed in the data query process, the schemes in [23,24,25,26,27] have been proposed to solve this problem.

Ghinita et al. [24] and Paulet et al. [25] employed the oblivious transfer to hide query location in the data query process, but the communication cost of schemes [24,25] is directly proportional to the data dimension. Zhu et al. [25,26] utilized an improved homomorphic encryption technology to protect the query location in location-based services, but it do not support scenarios with a high vehicle density. Kong et al. [23] utilized the proxy re-encryption technique to hide the query location, but it does not support queries of whole network sensory data during the data query phase.

To sum up, from the review above, the available data aggregation schemes [16,17,18,19,20,21,22,23] either fail to determine the number of data reports produced in each data dimension or bring heavy computation and communication costs. In addition, the communication costs of the existing schemes [23,24,25,26,27] are either directly proportional to the data dimension or bring heavy communication costs in the data query process.

To address the issues above, we propose an EP${}^{2}$DS scheme for fog-assisted vehicular sensor networks, which can not only reduce the computation and communication costs, but also calculate the average sensory data in each road segment. Additionally, the proposed EP${}^{2}$DS scheme can query the road conditions of potential moving routes without disclosing the query location.

## 3. Background

### 3.1. System Model

**Figure 1:**
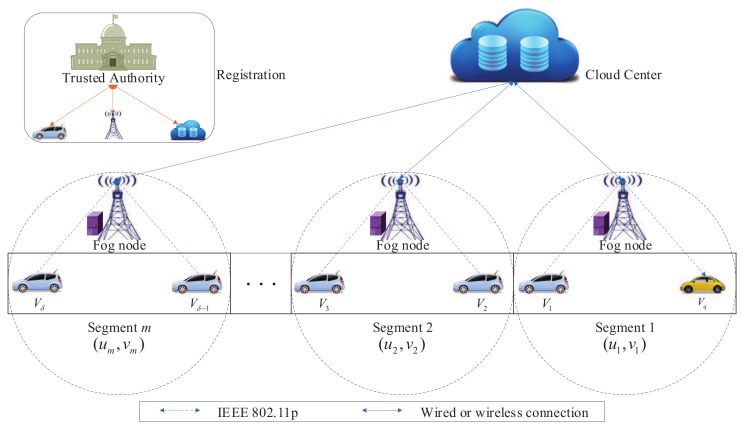
System model.

The system model is presented in Figure 1, which is composed of five entities: trusted authority (TA), cloud center (CC), the data collection vehicle $V_{i}$(i=1,2,···,δ), fog node $FN_{j}$(j=1,2,···,n), and the data query vehicle $V_{q}$. The road area is divided into *m* segments, and each segment *k*(k=1,2,···,m) is represented by a unique two-dimensional identifier $\left(u_{k},v_{k}\right)$, approximating of the location coordinates [23]. As to readability, the definitions of notations employed in this study are illustrated in Table 1.

The wireless connections between the vehicles and the fog nodes are brought about by the Institute of Electrical and Electronics Engineers (IEEE) 802.11p standard [29]. The connections between the fog nodes and CC are achieved via either the wired links or other links with low transmission delay and high bandwidth.

TA: A fully trusted entity, which is responsible for the management of the security parameters for the system and the registration of the cloud center, fog nodes, and vehicles, and periodically updates the system information.

CC: An honest-but-curious entity, which is responsible for providing centralized control with powerful storage and computing capabilities from a remote location. In addition, it can perform computational analytics from data reports uploaded by the fog nodes, and distribute data to all fog nodes for further sharing with vehicles [30].

$V_{i}$: It is equipped with smart sensors, periodically formatting a data report from the collected sensory data and uploading the data report towards the fog node.

$FN_{j}$: This consists of a road side unit and an edge server [13], and aggregates the data reports uploaded by the data collection vehicles under its communication range and transmits the aggregated data report towards CC. Meanwhile, each fog node manages one or more segments, and can assist in sharing the sensory data to the query vehicle [31].

$V_{q}$: To choose an optimal route, $V_{q}$ usually sends a query report to the fog node, then the fog node returns a response report to $V_{q}$.

In our system model, we assume the fog node is honest-but-curious, i.e., it is able to correctly execute the operations defined in the protocol; however, it also can try to violate the privacy of the vehicle through analyzing the vehicle’s data report and query report; meanwhile, we assume neither the fog nodes nor the query vehicles can collude with each other in the proposed EP${}^{2}$DS scheme. Additionally, we assume there exists an attacker, which can eavesdrop on the data transmission and launch attacks.

### 3.2. Security Requirement

The following security requirements should be achieved.

**Authentication and data integrity**: The proposed EP${}^{2}$DS scheme should guarantee that any reports are not modified during the transmission process, and can detect any modification of the reports; moreover, any entity in F-VSNs should be able to be authenticated to ensure the reliability of the data source.

**Confidentiality**: To ensure the privacy of sensory data, the proposed EP${}^{2}$DS scheme should provide confidentiality, i.e., no attacker can obtain the sensory data from data report.

**Location privacy preservation**: To protect vehicle’s query location, it is important not to disclose the query location to fog nodes that provide location-based services in the data query process.

**Identity privacy preservation**: Apart from the TA, any entities should not trace or recognize the identity of the data collection vehicle by analyzing the received data reports.

**Traceability**: TA should be able to reveal the identity of the malicious vehicle uploading the bogus data report.

**Unlinkability**: Apart from the TA, neither fog nodes nor the malicious vehicles can determine whether the two data reports are from the same vehicle.

**Resistance to attacks**: The proposed EP${}^{2}$DS scheme should be able to withstand various popular attacks such as the modification attack, replay attack, impersonation attack, and man-in-the-middle attack.

### 3.3. Elliptic Curve

Let $F_{p}$ be a finite field with a prime number *p*. The elliptic curve *E* over $F_{p}$ defined as the set of all points (x,y) meeting $y^{2}=x^{3}+ax+b\;\mathrm{mod}\;p$, where $4a^{3}+27b^{2}\neq 0$ and $a,b\in F_{p}$ [32,33].

An infinity point *O*, and other points on *E*, form an additive cyclic group G with the order *q* and generator *P*. Let P∈G and $k\in Z_{q}^{*}$, the scalar multiplication over G is described as kP=P+P+···+P (*k* times).

### 3.4. Security Assumption

**ECDL problem** [34,35]: Given two elements P,Q∈G, the ECDL problem is to find an integer $x\in Z_{q}^{*}$ such that Q=xP.

**ECDL assumption** [34,35]: It is hard for any probabilistic polynomial-time algorithm to solve ECDL problem with non-negligible probability.

## 4. The Proposed Scheme

**Figure 2:**
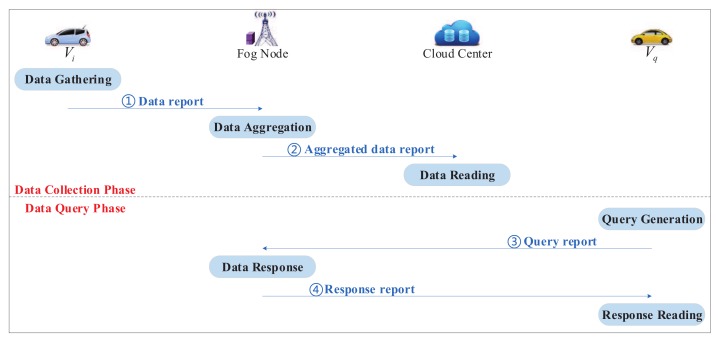
Data flows in the data collection and data query phases.

The proposed EP${}^{2}$DS scheme includes system initialization, registration, data collection, and data query phases. Note that the data flows in the data collection and data query phases are shown in Figure 2.

### 4.1. System Initialization

TA produces all system parameters through executing the following steps.

(1) TA randomly chooses a large prime number *p*, and selects a non-singular elliptic curve *E* defined by $y^{2}=x^{3}+ax+b\;\mathrm{mod}\;p$, where $a,b\in F_{p}$.
(2) TA picks a group G of *E* with the prime order *q* and a generator *P*.
(3) TA randomly chooses $s\in Z_{q}^{*}$ as its master key and computes its public key $P_{pub}=sP$.
(4) TA chooses eight one-way hash functions $H_{i}:{\left\{0,1\right\}}^{*}\to Z_{q}^{*}$, i=1,2,···,7, $H_{8}:{\left\{0,1\right\}}^{*}\to \in {\left\{0,1\right\}}^{|d|-1}$.
(5) TA chooses a super-increasing sequence $\vec{a}=\left(a_{1},a_{2},\cdot \cdot \cdot ,a_{m}\right)$, such that $\sum_{k=1}^{m}a_{k}3n\delta d<q$, $\sum_{k=1}^{i-1}a_{k}3n\delta d<a_{i}$ (i=1,2,···,m), where $a_{1},a_{2},\cdot \cdot \cdot ,a_{m}$ are large prime numbers and *d* is the maximum value of the data. Then, TA assigns prime number $a_{k}$ towards segment *k*.
(6) TA publishes the system parameters $\{p,q,G,P,P_{pub},$ $H_{1},H_{2},H_{3},H_{4},H_{5},H_{6},H_{7},H_{8},\vec{a}\}$.

### 4.2. Registration

All vehicles, fog nodes, and cloud centers register with TA.

#### 4.2.1. $V_{i}$ Registers with TA

(1) $V_{i}$ sends the identity $ID_{i}$ to the TA in secure channel.
(2) After confirming the identity $ID_{i}$, TA randomly chooses $w_{i}\in Z_{q}^{*}$ and computes
  $$PID_{i,1}=w_{i}P,PID_{i,2}=ID_{i}⊕H_{1}\left(w_{i}P_{pub},t_{i}\right),$$
  and sets $PID_{i}=\left\{PID_{i,1},PID_{i,2},t_{i}\right\}$, where $t_{i}$ represents the valid period of $PID_{i}$.
(3) TA randomly chooses $r_{i}\in Z_{q}^{*}$ and computes
  $$R_{i}=r_{i}P,x_{i}=r_{i}+sH_{2}\left(PID_{i},R_{i},P_{pub}\right).$$
(4) TA randomly chooses a sharing key $\varphi \in {\left\{0,1\right\}}^{|d|-1}$, and transmits the pseudo identity $PID_{i}$, the private key $\left(x_{i},R_{i}\right)$ and the sharing key φ to $V_{i}$ in a secure channel.

#### 4.2.2. $FN_{j}$ Registers with TA

(1) $FN_{j}$ sends the identity $ID_{FN_{j}}$ to the TA in a secure channel.
(2) TA randomly chooses $r_{FN_{j}}\in Z_{q}^{*}$ and computes
  $$R_{FN_{j}}=r_{FN_{j}}P,x_{FN_{j}}=r_{FN_{j}}+sH_{3}\left(ID_{FN_{j}},R_{FN_{j}},P_{pub}\right).$$
(3) TA sends the private key $\left(x_{FN_{j}},R_{FN_{j}}\right)$ to $FN_{j}$ in a secure channel.

#### 4.2.3. CC Registers with TA

(1) TA randomly chooses $x\in Z_{q}^{*}$ and computes $P_{cc}=xP$.
(2) TA sends the private key *x* and public key $P_{cc}$ to CC in a secure channel.

### 4.3. Data Collection

The data collection phase includes three processes: data gathering, data aggregation, and data reading.

#### 4.3.1. Data Gathering

$V_{i}$ gathers sensory data in a short period of time, e.g., every five minutes: (i) if there is a sensory data obtained at road segment *k* under $FN_{j}$, i.e., $d_{i,k}^{j}>0$, then $e_{i,k}^{j}=1$; (ii) if there is no sensory data obtained at road segment *k* under $FN_{j}$, i.e., $d_{i,k}^{j}=0$, then $e_{i,k}^{j}=0$.

$V_{i}$ produces a data report through executing the following steps:

(1) $V_{i}$ formats $\left(d_{i,1}^{j},d_{i,2}^{j},\cdot \cdot \cdot ,d_{i,m}^{j}\right)$ and $\left(e_{i,1}^{j},e_{i,2}^{j},\cdot \cdot \cdot ,e_{i,m}^{j}\right)$ into $d_{i}^{j}=\sum_{k=1}^{m}a_{k}\left(d_{i,k}^{j}+\varphi \right)$ and $e_{i}^{j}=\sum_{k=1}^{m}a_{k}\left(e_{i,k}^{j}+\varphi \right)$.
(2) $V_{i}$ randomly selects $r_{i}^{j}$, $s_{i}^{j}\in Z_{q}^{*}$ and computes
  $$A_{i}^{j}=r_{i}^{j}P,B_{i}^{j}=d_{i}^{j}P+r_{i}^{j}P_{cc},C_{i}^{j}=s_{i}^{j}P,D_{i}^{j}=e_{i}^{j}P+s_{i}^{j}P_{cc}.$$
(3) $V_{i}$ randomly picks $l_{i}^{j}\in Z_{q}^{*}$ and calculates
  $$L_{i}^{j}=l_{i}^{j}P,\sigma_{i}^{j}=x_{i}+l_{i}^{j}H_{4}\left(PID_{i},R_{i},A_{i}^{j},B_{i}^{j},C_{i}^{j},D_{i}^{j},L_{i}^{j},T_{i}^{j}\right),$$
  where $T_{i}^{j}$ is current timestamp.
(4) $V_{i}$ transmits the data report $DR_{i}^{j}=\{PID_{i},R_{i},A_{i}^{j},B_{i}^{j},$ $C_{i}^{j},D_{i}^{j},L_{i}^{j},\sigma_{i}^{j},T_{i}^{j}\}$ towards $FN_{j}$, as shown in Figure 2 (①).

#### 4.3.2. Data Aggregation

Supposing *w* vehicles $\{V_{1},V_{2},\cdot \cdot \cdot ,V_{w}\}$ upload the data reports $\left\{DR_{1}^{j},DR_{2}^{j},\cdot \cdot \cdot ,DR_{w}^{j}\right\}$ to $FN_{j}$, where w≤δ. $FN_{j}$ can aggregate data reports through executing the following steps:

(1) $FN_{j}$ checks whether $t_{i}$ is valid and $T_{i}^{j}$ is fresh for each i=1,2,···,w. If $t_{i}$ is not valid or $T_{i}^{j}$ is not fresh, $DR_{i}^{j}$ will be rejected. Otherwise, $FN_{j}$ performs the batch verification using small exponent test [36]. $FN_{j}$ randomly selects a set of small numbers $\theta_{1}^{j},\theta_{2}^{j},\cdot \cdot \cdot ,\theta_{w}^{j}\in \left[1,2^{w}\right]$ and checks whether the following equation holds
  $$\begin{array}{c} \sum_{i=1}^{w}\theta_{i}^{j}\sigma_{i}^{j}P=\sum_{i=1}^{w}\theta_{i}^{j}R_{i}+\sum_{i=1}^{w}\theta_{i}^{j}H_{2}\left(PID_{i},R_{i},P_{pub}\right)P_{pub}\\ \;\;\;\;\;\;+\sum_{i=1}^{w}\theta_{i}^{j}H_{4}\left(PID_{i},R_{i},A_{i}^{j},B_{i}^{j},C_{i}^{j},D_{i}^{j},L_{i}^{j},T_{i}^{j}\right)L_{i}^{j}. \end{array}$$
  If it does hold, $FN_{j}$ computes
  $$A^{j}=\sum_{i=1}^{w}A_{i}^{j},B^{j}=\sum_{i=1}^{w}B_{i}^{j},C^{j}=\sum_{i=1}^{w}C_{i}^{j},D^{j}=\sum_{i=1}^{w}D_{i}^{j}.$$
(2) $FN_{j}$ randomly picks $l^{j}\in Z_{q}^{*}$ and calculates
  $$L^{j}=l^{j}P,\sigma^{j}=x_{FN_{j}}+l^{j}H_{5}\left(ID_{FN_{j}},R_{FN_{j}},A^{j},B^{j},C^{j},D^{j},L^{j},T^{j}\right),$$
  where $T^{j}$ is current timestamp.
(3) $FN_{j}$ transmits the aggregated data report $ADR^{j}=\left\{ID_{FN_{j}},R_{FN_{j}},A^{j},B^{j},C^{j},D^{j},L^{j},\sigma^{j},T^{j}\right\}$ towards CC, as shown in Figure 2 (②).

#### 4.3.3. Data Reading

After receiving $\left\{ADR^{1},ADR^{2},\cdot \cdot \cdot ,ADR^{n}\right\}$ from $\{FN_{1},$ $FN_{2},\cdot \cdot \cdot ,FN_{n}\}$ respectively, CC executes the following steps:

(1) CC checks whether $T^{j}$ is fresh for each j=1,2,···,n. If $T^{j}$ is not fresh, $ADR^{j}$ will be rejected. Otherwise, CC randomly chooses a set of small numbers $\theta^{1},\theta^{2},\cdot \cdot \cdot ,\theta^{n}\in \left[1,2^{n}\right]$ and performs the batch verification using small exponent test [36]. CC verifies whether the following equation holds
  $$\begin{array}{c} \sum_{j=1}^{n}\theta^{j}\sigma^{j}P=\sum_{j=1}^{n}\theta^{j}R_{FN_{j}}+\sum_{j=1}^{n}\theta^{j}H_{3}\left(ID_{FN_{j}},R_{FN_{j}},P_{pub}\right)P_{pub}\\ \;\;\;\;\;\;\;\;+\sum_{j=1}^{n}\theta^{j}H_{5}(ID_{FN_{j}},R_{FN_{j}},A^{j},B^{j},C^{j},D^{j},L^{j},T^{j})L^{j}. \end{array}$$
  If it does hold, CC calculates
  $$\Phi =\sum_{j=1}^{n}B^{j}-x\cdot \sum_{j=1}^{n}A^{j},\Delta =\sum_{j=1}^{n}D^{j}-x\cdot \sum_{j=1}^{n}C^{j}.$$
  By solving the discrete log of Φ and Δ with the base *P*, utilizing the Pollard’s lambda algorithm [37], CC can obtain
  $$\mu =\sum_{j=1}^{n}\sum_{i=1}^{w}\left(\varphi +d_{i}^{j}\right),\nu =\sum_{j=1}^{n}\sum_{i=1}^{w}\left(\varphi +e_{i}^{j}\right).$$
(2) CC distributes μ and ν to all fog nodes $\left\{FN_{1},FN_{2},\cdot \cdot \cdot ,FN_{n}\right\}$ for further sharing with vehicles.

### 4.4. Data Query

The data query vehicle $V_{q}$ intends to query the data captured at segment *c* with the identifier $\left(u_{c},v_{c}\right)$ at the $FN_{j}$. The phase includes three processes: query generation, data response, and response reading.

#### 4.4.1. Query Generation

(1) $V_{q}$ selects two random numbers $r_{q}^{j},s_{q}^{j}\in Z_{q}^{*}$ and calculates
  $$E_{q}^{j}=r_{q}^{j}P,F_{q}^{j}=u_{c}P+x_{q}E_{q}^{j},G_{q}^{j}=s_{q}^{j}P,H_{q}^{j}=v_{c}P+x_{q}G_{q}^{j}.$$
(2) $V_{q}$ randomly picks $l_{q}^{j}\in Z_{q}^{*}$ and calculates
  $$L_{q}^{j}=l_{q}^{j}P,\sigma_{q}^{j}=x_{q}+l_{q}^{j}H_{6}\left(PID_{q},R_{q},E_{q}^{j},F_{q}^{j},G_{q}^{j},H_{q}^{j},L_{q}^{j},T_{q}^{j}\right),$$
  where $T_{q}^{j}$ is the current timestamp.
(3) $V_{q}$ transmits the query report $QR_{q}^{j}=\{PID_{q},R_{q},E_{q}^{j},F_{q}^{j},$ $G_{q}^{j},H_{q}^{j},L_{q}^{j},\sigma_{q}^{j},T_{q}^{j}\}$ towards $FN_{j}$, as shown in Figure 2 (③).

#### 4.4.2. Data Response

(1) After receiving $QR_{q}^{j}$, $FN_{j}$ checks whether $t_{q}$ is valid and $T_{q}^{j}$ is fresh. If $t_{q}$ is not valid or $T_{q}^{j}$ is not fresh, $QR_{q}^{j}$ will be rejected. Otherwise, $FN_{j}$ verifies whether the following equation holds
  $$\begin{array}{c} \sigma_{q}^{j}P=R_{q}+H_{2}\left(PID_{q},R_{q},P_{pub}\right)P_{pub}+H_{6}\left(PID_{q},R_{q},E_{q}^{j},F_{q}^{j},G_{q}^{j},H_{q}^{j},L_{q}^{j},T_{q}^{j}\right)L_{q}^{j}. \end{array}$$
  If it does hold, $FN_{j}$ selects two random numbers $t_{q}^{j},\varphi_{q}^{j}\in Z_{q}^{*}$ and calculates
  $$\;J_{q}^{j}=t_{q}^{j}E_{q}^{j}+\varphi_{q}^{j}G_{q}^{j},K_{q}^{j}=t_{q}^{j}F_{q}^{j}+\varphi_{q}^{j}H_{q}^{j},$$
  $$M_{q}^{j}=\mu +\sum_{k=1}^{m}a_{k}H_{8}\left(t_{q}^{j}u_{k}+\varphi_{q}^{j}v_{k}\right),N_{q}^{j}=\nu +\sum_{k=1}^{m}a_{k}H_{8}\left(t_{q}^{j}u_{k}+\varphi_{q}^{j}v_{k}\right).$$
(2) $FN_{j}$ randomly picks ${\hat{l}}_{q}^{j}\in Z_{q}^{*}$ and calculates
  $${\hat{L}}_{q}^{j}={\hat{l}}_{q}^{j}P,{\hat{\sigma }}_{q}^{j}=x_{FN_{j}}+{\hat{l}}_{q}^{j}H_{7}\left(ID_{FN_{j}},R_{FN_{j}},J_{q}^{j},K_{q}^{j},M_{q}^{j},N_{q}^{j},{\hat{L}}_{q}^{j},{\hat{T}}_{q}^{j}\right),$$
  where ${\hat{T}}_{q}^{j}$ is the current timestamp.
(3) $FN_{j}$ transmits the response report $RR_{q}^{j}=\{ID_{FN_{j}},$ $R_{FN_{j}},J_{q}^{j},K_{q}^{j},M_{q}^{j},N_{q}^{j},{\hat{L}}_{q}^{j},{\hat{\sigma }}_{q}^{j},{\hat{T}}_{q}^{j}\}$ towards $V_{q}$, as shown in Figure 2 (④).

#### 4.4.3. Response Reading

(1) After receiving $RR_{q}^{j}$, $V_{q}$ checks whether ${\hat{T}}_{q}^{j}$ is fresh. If ${\hat{T}}_{q}^{j}$ is not fresh, $RR_{q}^{j}$ will be rejected. Otherwise, $V_{q}$ verifies whether the following equation holds
  $$\begin{array}{c} {\hat{\sigma }}_{q}^{j}P=R_{FN_{j}}+H_{3}\left(ID_{FN_{j}},R_{FN_{j}},P_{pub}\right)P_{pub}+H_{7}\left(ID_{FN_{j}},R_{FN_{j}},J_{q}^{j},K_{q}^{j},M_{q}^{j},N_{q}^{j},{\hat{L}}_{q}^{j},{\hat{T}}_{q}^{j}\right){\hat{L}}_{q}^{j}. \end{array}$$
  If it does hold, $V_{q}$ calculates
  $$\Lambda =K_{q}^{j}-x_{q}\cdot J_{q}^{j}.$$
  By solving the discrete log of Λ with the base *P*, utilizing the Pollard’s lambda algorithm [37], $V_{q}$ can obtain $\beta_{c}=H_{8}\left(t_{q}^{j}u_{c}+\varphi_{q}^{j}v_{c}\right)$.
(2) By calling the Algorithm 1, $V_{q}$ can achieve the average sensing data ${\bar{d}}_{c}$ captured at segment *c*.

**Algoruthm 1** Recovery ${\bar{d}}_{c}$ captured at segment *c***Input:** $\left(a_{1},a_{2},\cdot \cdot \cdot ,a_{m}\right)$, $\beta_{c}$, φ, δ, $M_{q}^{j}$ and $N_{q}^{j}$
**Output: **
${\bar{d}}_{c}$

**begin:**
      **set**
$X_{1}=M_{q}^{j}$, $X_{2}=N_{q}^{j}$;
      **for**
k=m to *c*
**do**
             $d_{k}=\frac{X_{1}-X_{1}\;\mathrm{mod}\;a_{k}}{a_{k}}$, $e_{k}=\frac{X_{2}-X_{2}\;\mathrm{mod}\;a_{k}}{a_{k}}$;
            $X_{1}=X_{1}\;\mathrm{mod}\;a_{k}$, $X_{2}=X_{2}\;\mathrm{mod}\;a_{k}$;
      **return**
${\bar{d}}_{c}=\frac{d_{c}-\beta_{c}-\delta \varphi }{e_{c}-\beta_{c}-\delta \varphi }$.

**end**


## 5. Security

This section depicts the security proof of the proposed EP${}^{2}$DS scheme in the random oracle model. Additionally, a security evaluation and comparison on the proposed EP${}^{2}$DS scheme and schemes of [17,19,23,25,26] is conducted.

### 5.1. Security Model

The security model of the proposed EP${}^{2}$DS scheme can be found in the Appendix A.

### 5.2. Security Proof

The security proof of the proposed EP${}^{2}$DS scheme can be found in the Appendix B.

### 5.3. Analysis and Comparison of Security Requirement

**Authentication and data integrity**: Based on Theorem 2, no polynomial-time attacker is able to fake a valid data report owing to the ECDL assumption. Therefore, authentication and data integrity can be ensured in the proposed EP${}^{2}$DS scheme.

**Confidentiality**: Based on Theorem 1, without the cloud center’s private key *x*, any attacker is unable to compute the sensing data $\mu =\sum_{j=1}^{n}\sum_{i=1}^{w}\left(\varphi +d_{i}^{j}\right)$ and $\nu =\sum_{j=1}^{n}\sum_{i=1}^{w}\left(\varphi +e_{i}^{j}\right)$, and thus confidentiality can be ensured in the proposed EP${}^{2}$DS scheme.

**Location privacy preservation**: Based on Theorem 1, without the the data query vehicle’s private key $x_{q}$, no attacker can obtain the query location $\left(u_{c},v_{c}\right)$ from $\{E_{q}^{j}=r_{q}^{j}P$, $F_{q}^{j}=u_{c}P+x_{q}E_{q}^{j}$, $G_{q}^{j}=s_{q}^{j}P$, $H_{q}^{j}=v_{c}P+x_{q}G_{q}^{j}\}$, and hence the location privacy can be guaranteed in the proposed EP${}^{2}$DS scheme.

**Identity privacy preservation**: On the basis of the proposed EP${}^{2}$DS scheme, the identity $ID_{i}$ of $V_{i}$ is only contained in the pseudo identity $PID_{i}=\left\{PID_{i,1},PID_{i,2},t_{i}\right\}$, where $PID_{i,1}=w_{i}P$, $PID_{i,2}=ID_{i}⊕H\left(w_{i}P_{pub},t_{i}\right)$ and $P_{pub}=sP$. To extract the identity $ID_{i}$ of $V_{i}$, the attacker has to compute $ID_{i}=PID_{i,2}⊕H\left(s\cdot PID_{i,2},t_{i}\right)$. However, it is impossible to solve $w_{i}\cdot s\cdot P$ for any attacker to obtain $ID_{i}$ without knowing $w_{i}$ and *s*. Therefore, the identity privacy is guaranteed in the proposed EP${}^{2}$DS scheme.

**Traceability**: In accordance with the proposed EP${}^{2}$DS scheme, TA can adopt its own master key *s* to calculate $ID_{i}=PID_{i,2}⊕H\left(s\cdot PID_{i,2},t_{i}\right)$, and find out the identity $ID_{i}$ of $V_{i}$ from the pseudo identity $PID_{i}$ involved in the data report, with the proposed EP${}^{2}$DS scheme satisfying the traceability.

**Unlinkability**: On the basis of the proposed EP${}^{2}$DS scheme, the data reports generated by any vehicle are random, and any attacker cannot link the two data reports sent by the same vehicle, with the proposed EP${}^{2}$DS scheme realizing the traceability.

**Resistance to attacks**: The proposed EP${}^{2}$DS scheme is able to withstand the networks attacks in the following:

- **Modification attack:** Based on Theorem 2, any polynomial attacker is unable to forge a valid data report with modification on data reports found.
- **Replay attack:** On the basis of the proposed EP${}^{2}$DS scheme, the timestamp is contained in the data report. By examining freshness of the timestamp, the verifier is able to bear any replay attacks.
- **Impersonation attack:** From Theorem 2, no attacker can fabricate a legal data report without vehicle’s private key.
- **Man-in-the-middle attack:** The analysis of the modification attack shows that any modification of the data reports on transmission is able to be found.

Security comparisons of schemes [17,19,23,25,26] and the proposed EP${}^{2}$DS scheme are displayed in Table 2. S1, S2, S3, S4, S5, S6, S7, S8, S9, and S10 are used to represent authentication and data integrity, confidentiality, location privacy preservation, identity privacy preservation, traceability, unlinkability, the modification attack, the replay attack, the impersonation attack, and the man-in-the-middle attack, respectively.

In accordance with Table 2, Rabieh et al.’s scheme [17] is able to provide location privacy preservation, identity privacy preservation, and traceability. Sun et al.’s scheme [19] cannot achieve location privacy preservation. Kong et al.’s scheme [23] cannot achieve identity privacy preservation, traceability, the replay attack, and the man-in-the-middle attack. Paulet et al.’s scheme [25] cannot achieve authentication and data integrity, identity privacy preservation, traceability, the modification attack, the replay attack, the impersonation attack, and the man-in-the-middle attack. Zhu et al.’s scheme [26] cannot achieve identity privacy preservation and traceability, the replay attack, and the man-in-the-middle attack. In contrast, all security requirements are able to be satisfied in the proposed EP${}^{2}$DS scheme.

## 6. Performance Evaluation

We analyze the computation and communication costs of these schemes [17,19,23,25,26] and the proposed EP${}^{2}$DS scheme, and evaluate their performance.

To realize a fair comparison, we compare these schemes [17,19,23,25,26] with the proposed EP${}^{2}$DS scheme under the 80-bit security level [38]. Regarding the pairing-based schemes [17,19,23,25,26], we choose a bilinear pairing $e:G_{1}\times G_{1}\to G_{2}$, where $G_{1}$ is an additive group defined by the generator *P* with order *q* on the super singular elliptic curve $E:y^{2}=x^{3}+x\;\mathrm{mod}\;p$ with the embedding degree 2, *q* is 160-bit Solinas prime number and *p* is 512-bit primer number meeting q·12·r=p+1. With regard to the proposed EP${}^{2}$DS scheme, we pick a group G, where G is produced by the generator *P* with the order *q* on an elliptic curve $E:y^{2}=x^{3}+ax+b\;\mathrm{mod}\;p$ with a prime order *q*, where *q*, *p* are 160 bits prime number and a=-3, *b* is 160-bits random prime number.

The running time of the operations is able to be derived by making use of the MIRACL Crypto SDK [39]. We run the experiment on a 64-bit Windows 10 operating system with 2.53 GHz, an i7 CPU and 4 GB memory. Table 3 lists the average running time for these operations.

### 6.1. Computation Costs

The computation costs of the proposed EP${}^{2}$DS scheme and these schemes [17,19,23,25,26] are displayed in Table 4.

In the data collection phase, for Rabieh et al.’s scheme [17], $V_{i}$ requires running two multiplication operations in $G_{1}$ and two exponentiation operations in $G_{1}$, thus the total time is $2T_{m}+2T_{e}$ = 6.9164 ms. FN requires executing one multiplication operation in $G_{1}$, one exponentiation operation in $G_{1}$, and w+1 bilinear pairing operations in $G_{1}$, and thus the total time is $T_{m}+T_{e}+\left(w+1\right)T_{p}$ = 10.3092*w*+13.7674 ms. CC requires executing one exponentiation operation in $G_{1}$ and n+1 bilinear pairing operations in $G_{1}$, and hence the total time is $T_{e}+\left(n+1\right)T_{p}=10.3092n+2.0289$ ms.

For Sun et al.’s scheme [19], $V_{i}$ requires running two multiplication operations in $G_{1}$ and one exponentiation operation in $G_{1}$ and one map to point hash function operation, thus the total time is $2T_{m}+T_{e}+T_{h}$ = 15.1967 ms. FN requires executing w+3 multiplication operations in $G_{1}$ and four bilinear pairing operations in $G_{1}$, so the total time is $\left(w+3\right)T_{m}+4T_{p}$ = 1.4293*w* +45.5247 ms. CC requires executing one multiplication operation in $G_{1}$, *n* exponentiation operations in $G_{1}$ and two multiplication operations in $G_{1}$, and hence the total time is $T_{m}+nT_{e}+2T_{p}=2.0289n+11.7385$ ms.

For Kong et al.’s scheme [23], $V_{i}$ requires running four multiplication operations in $Z_{n^{2}}$ and four exponentiation operations in $Z_{n^{2}}$, thus the total time is $4T_{m}+4T_{e}$ = 13.8328 ms. FN requires executing 2w multiplication operations in $G_{1}$, so the total time is $2wT_{m}$ = 2.8586*w* ms. CC requires executing 6n multiplication operations in $G_{1}$ and 4n exponentiation operations in $G_{1}$, and hence the total time is $6nT_{m}+4nT_{e}=16.6914n$ ms.

For the proposed EP${}^{2}$DS scheme, $V_{i}$ needs to run five scalar multiplication operations in G, and therefore the total time is $5T_{sm}$ = 1.9255 ms. FN requires executing w+3 scalar multiplication operations in G; accordingly, the total time is $\left(w+3\right)T_{sm}$ = 0.3851*w*+1.1553 ms. CC requires executing n+3 scalar multiplication operations in G and two solving the DL operations; therefore, the total time is $\left(n+3\right)T_{sm}+2T_{log}$ = 0.3851*n*+2.4429 ms.

In the data query phase, for Kong et al.’s scheme [23], $V_{q}$ requires running ten multiplication operations in $G_{1}$ and seven exponentiation operations in $G_{1}$, so the total time is $10T_{m}+7T_{e}$ = 28.4953 ms. FN needs to run nine multiplication operations in $G_{1}$ and seven exponentiation operations in $G_{1}$, the total time is thus $9T_{m}+7T_{e}$ = 27.0660 ms. For Paulet et al.’s scheme [25], $V_{q}$ requires running five multiplication operations in $G_{1}$ and nine exponentiation operations in $G_{1}$, the total time is thus $5T_{m}+9T_{e}$ = 25.4066 ms. FN needs to run 6m multiplication operations in $G_{1}$ and 8m+3 exponentiation operations in $G_{1}$, the total time is thus $6mT_{m}+\left(8m+3\right)T_{e}$ = 24.8070*m* +6.0867 ms.

For Zhu et al.’s scheme [26], $V_{q}$ requires running five exponentiation operations in $G_{1}$ and two bilinear pairing operation in $G_{1}$, the total time is thus $5T_{e}+2T_{p}$ = 30.7629 ms. FN needs to run four multiplication operations in $G_{1}$ and four bilinear pairing operation in $G_{1}$, the total time is thus $4T_{m}+4T_{p}$ = 46.9540 ms.

For the proposed EP${}^{2}$DS scheme, $V_{q}$ needs to run eleven scalar multiplication operations in G and two solving the DL operations, and hence the total time is $11T_{sm}+2T_{log}$ = 5.5237 ms. FN needs to run eight scalar multiplication operations in G, thus the total time is $8T_{sm}$ = 3.0808 ms.

**Figure 3:**
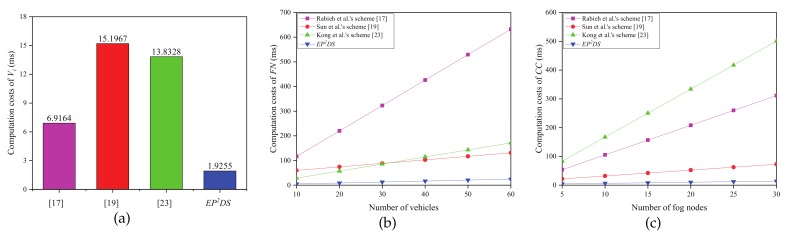
Computation costs in the data collection phase. (**a**) Computation costs of $V_{i};$ (**b**) Computation costs of FN vs. number of vehicles; (**c**) Computation costs of CC vs. number of FN.

Figure 3 clearly demonstrates the comparison result of computation costs in the data collection phase. Figure 3a shows that the computation costs of $V_{i}$ is 1.9255 ms, which decreases by 72.2%, 87.3%, and 86.1% compared with that by Rabieh et al.’s scheme [17], Sun et al.’s scheme [19], and Kong et al.’s scheme [23], respectively. As shown in Figure 3b, the computation costs of FN increase linearly with the number of vehicles, with the proposed EP${}^{2}$DS scheme having a lower slope compared with Rabieh et al.’s scheme [17], Sun et al.’s scheme [19], and Kong et al.’s scheme [23]. From Figure 3c, we can see that the computation costs of CC grows linearly with the number of fog nodes, and the proposed EP${}^{2}$DS scheme has a lower slope compared with Rabieh et al.’s scheme [17], Sun et al.’s scheme [19], and Kong et al.’s scheme [23].

**Figure 4:**
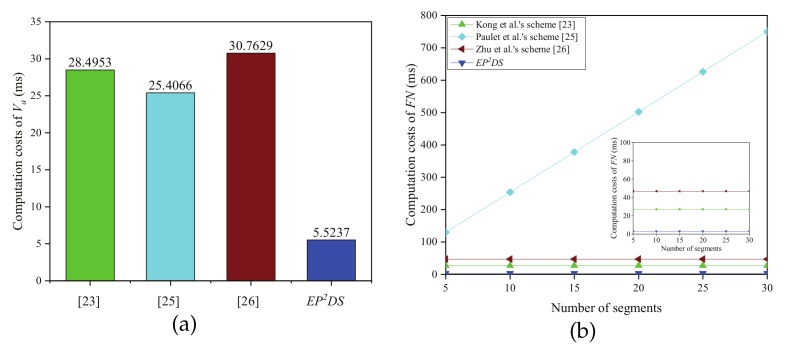
Computation costs in the data query phase. (**a**) Computation costs of $V_{q};$ (**b**) Computation costs of FN vs. number of segments.

Figure 4 clearly indicates the comparison result of the computation costs in the data query phase. From Figure 4a, we can know that the computation costs of $V_{q}$ in the proposed EP${}^{2}$DS scheme are 5.5237 ms, which decreases by 80.6%, 78.3%, and 82.0% compared with that by Kong et al.’s scheme [23], Paulet et al.’s scheme [25], and Zhu et al.’s scheme [26], respectively. Figure 4b shows the correlation between the computation cost of FN and the number of segments *m*, we can see that the computation cost of FN in the EP${}^{2}$DS scheme is the smallest compared with Kong et al.’s scheme [23], Paulet et al.’s scheme [25], and Zhu et al.’s scheme [26]. The computation costs of FN in the proposed EP${}^{2}$DS scheme are 3.0808 ms, which decreases by 88.6% and 93.4% compared with Kong et al.’s scheme [23] and Zhu et al.’s scheme [26]. Furthermore, unlike Paulet et al.’s scheme [25], the computation cost of FN in the EP${}^{2}$DS scheme does not increase with the number of segments *m*.

### 6.2. Communication Costs

The communication costs of the proposed EP${}^{2}$DS scheme and these schemes [17,19,23,25,26], are evaluated in this subsection. We mainly consider the data report size, query report size, and response report size. As mentioned above, the lengths of the elements in G, $Z_{q}^{*},Z_{n}$, and $Z_{n^{2}}$ are 160 bits (20 bytes), 160 bits (20 bytes), 1024 bits (128 bytes), and 2048 bits (256 bytes), respectively, assuming that the length of timestamp and identity are 32 bits (4 bytes). The comparison results of communication costs are illustrated in Table 5.

In the data collection phase, for Rabieh et al.’s scheme [17], the data report size is 260 bytes, as

$$\begin{array}{c} |C_{v}\left|+|TS|+\right|\alpha_{v}|=128+4+128=260\;\mathrm{bytes}. \end{array}$$

For Sun et al.’s scheme [19], the data report size is 516 bytes, as

$$\begin{array}{c} |S_{c}\left|+\right|SignC_{i}\left|+\right|t_{i}|=256+256+4=516\;\mathrm{bytes}. \end{array}$$

For Kong et al.’s scheme [23], the data report size is 1152 bytes, as

$$\begin{array}{c} |C_{i,1}\left|+\right|C_{i,2}\left|+\right|C_{i,3}\left|+\right|C_{i,4}|+|MAC_{i}|=256+256+256+256+128=1152\;\mathrm{bytes}. \end{array}$$

For the proposed EP${}^{2}$DS scheme, the data report size is 172 bytes, as

$$\begin{array}{c} \;|PID_{i}\left|+\right|R_{i}\left|+\right|A_{i}^{j}\left|+\right|B_{i}^{j}\left|+\right|C_{i}^{j}\left|+\right|D_{i}^{j}\left|+\right|L_{i}^{j}\left|+\right|\sigma_{i}^{j}\left|+\right|T_{i}^{j}|\\ =28+20+20+20+20+20+20+20+4+4=172\;\mathrm{bytes}. \end{array}$$

In the data query phase, for Kong et al.’s scheme [23], the query report size is 1152 bytes, as

$$\begin{array}{c} |C_{a,1}\left|+\right|C_{a,2}\left|+\right|C_{a,3}\left|+\right|C_{a,4}|+|MAC_{a}|=256+256+256+256+128=1152\;\mathrm{bytes}. \end{array}$$

The response report size is 1664 bytes, as

$$\begin{array}{c} \;|C_{r,1}\left|+\right|C_{r,2}\left|+\right|C_{r,3}\left|+\right|C_{r,4}\left|+\right|C_{r,5}\left|+\right|C_{r,6}|+|MAC_{r}|\\ =256+256+256+256+256+256+128=1664\;\mathrm{bytes}. \end{array}$$

For Paulet et al.’s scheme [25], the query report size is 256 bytes, as

$$\begin{array}{c} |C_{1}\left|+\right|C_{2}|=128+128=256\;\mathrm{bytes}. \end{array}$$

The response report size is 256*m*+128 bytes, as

$$\begin{array}{c} \;|C_{1,1}^{{}^{\prime }}\left|+\right|C_{1,2}^{{}^{\prime }}\left|+\cdot \cdot \cdot +\right|C_{1,m}^{{}^{\prime }}\left|+\right|C_{2,1}^{{}^{\prime }}\left|+\right|C_{2,2}^{{}^{\prime }}\left|\cdot \cdot \cdot +\right|C_{2,m}^{{}^{\prime }}\left|+|\gamma \right|\\ =128m+128m+128=256m+128\;\mathrm{bytes}. \end{array}$$

For Zhu et al.’s scheme [26], the query report size is 324 bytes, as

$$\begin{array}{c} |ID_{LBS}\left|+\right|E_{LQR}\left|+\right|U_{i}|+|TS|+|Sig_{i}|=4+256+256+4+256=324\;\mathrm{bytes}. \end{array}$$

The response report size is 320 bytes, as

$$\begin{array}{c} |E_{rq_{1}}\left(TRL\right)|+|ID_{cs}|+|TS|+|Sig_{cs}|=256+4+4+256=320\;\mathrm{bytes}. \end{array}$$

For the proposed EP${}^{2}$DS scheme, the query report size is 172 bytes, as

$$\begin{array}{c} \;|PID_{q}\left|+\right|R_{q}\left|+\right|A_{q}^{j}\left|+\right|B_{q}^{j}\left|+\right|C_{q}^{j}\left|+\right|D_{q}^{j}\left|+\right|L_{q}^{j}\left|+\right|\sigma_{q}^{j}\left|+\right|T_{q}^{j}|\\ =28+20+20+20+20+20+20+20+4=172\;\mathrm{bytes}. \end{array}$$

The response report size is 148 bytes, as

$$\begin{array}{c} \;|ID_{FN_{j}}\left|+\right|R_{FN_{j}}\left|+\right|J_{q}^{j}\left|+\right|K_{q}^{j}\left|+\right|M_{q}^{j}\left|+\right|N_{q}^{j}\left|+\right|{\hat{L}}_{q}^{j}\left|+\right|{\hat{\sigma }}_{q}^{j}\left|+\right|{\hat{T}}_{q}^{j}|\\ =4+20+20+20+20+20+20+20+4=148\;\mathrm{bytes}. \end{array}$$

**Figure 5:**
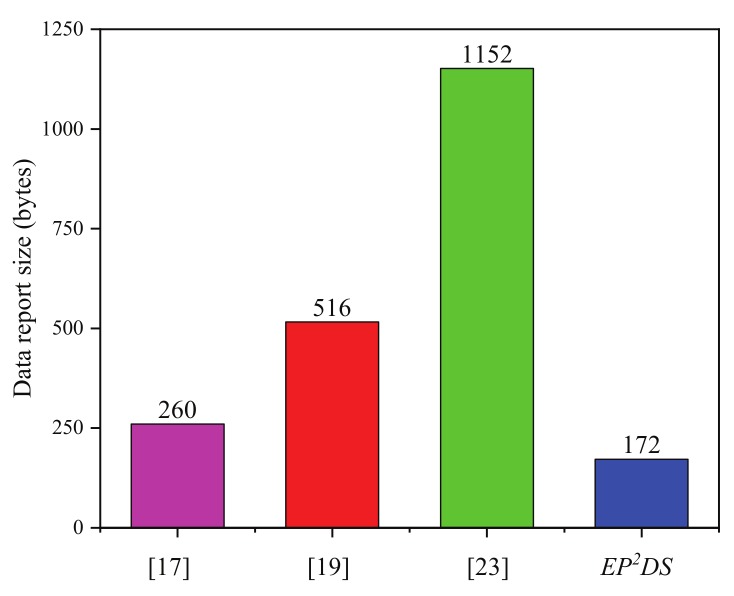
Comparison of the data report size.

The results from the comparison of communication costs in the data collection phase are illustrated in Figure 5. In terms of the data report size, the proposed EP${}^{2}$DS scheme requires 172 bytes, which is decreased by 33.8%, 66.7%, and 85.1% compared with that for Rabieh et al.’s scheme [17], Sun et al.’s scheme [19], and Kong et al.’s scheme [23], respectively.

**Figure 6:**
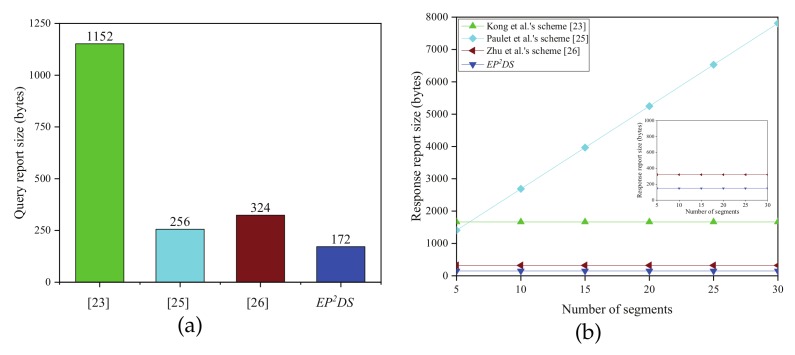
(**a**) Comparison of the query report size; (**b**) Comparison of the response report size.

The result from the comparison of communication costs in the data query phase is shown in Figure 6. Regarding the query report size, from Figure 6a, we can see that the proposed EP${}^{2}$DS scheme requires 172 bytes, a decrease of 85.1%, 32.8%, and 46.9% compared with that by Kong et al.’s scheme [23], Paulet et al.’s scheme [25], and Zhu et al.’s scheme [26], respectively. Figure 6b shows the correlation between the response report size and the number of segments *m*, and we can see that the response report size in the EP${}^{2}$DS scheme is the smallest compared with Kong et al.’s scheme [23], Paulet et al.’s scheme [25], and Zhu et al.’s scheme [26]. The proposed EP${}^{2}$DS scheme requires 148 bytes, which is decreased by 91.1% and 53.8% compared with that of Kong et al.’s scheme [23] and Zhu et al.’s scheme [26], respectively. Furthermore, unlike Paulet et al.’s scheme [25], the response report size in the EP${}^{2}$DS scheme does not increase with the number of segments *m*.

## 7. Conclusions

This paper proposes an efficient privacy-preserving data sharing scheme for fog-assisted vehicular sensor networks. Based on the super-increasing sequence, the proposed EP${}^{2}$DS scheme is able to format the data reports captured at different road segments into one report, while calculating the average sensory data in each road segment, greatly saving on the resources of communication and computation. Furthermore, by exploiting the modified oblivious transfer technology, the proposed EP${}^{2}$DS scheme also can query the road conditions of the potential moving route in the data query phase without disclosing the query location. Finally, an analysis of security displays that the proposed EP${}^{2}$DS scheme can satisfy all the requirements for security and privacy, with the performance evaluation suggesting that the proposed EP${}^{2}$DS scheme is more efficient in computation and communication costs compared to the existing schemes of [17,19,23,25,26]. Accordingly, the proposed EP${}^{2}$DS scheme is more appropriate for achieving data sharing in fog-assisted vehicular sensor networks. In future work, we will consider using blockchain technology to achieve decentralization and privacy protection.

## Figures and Tables

**Table 1:** Notations

Symbol	Definition
TA	Trusted authority
CC	Cloud center
$\left(s,P_{pub}\right)$	TA’s public key and private key
$\left(x,P_{cc}\right)$	CC’s public key and private key
$V_{i}$	The *i*-th data collection vehicle
$\left(ID_{i},PID_{i}\right)$	$V_{i}$’s real identity and pseudo identity
$\left(x_{i},R_{i}\right)$	$V_{i}$’s private key
$FN_{j}$	The *j*-th fog node
$ID_{j}$	$FN_{j}$’s identity
$\left(x_{FN_{j}},R_{FN_{j}}\right)$	$FN_{j}$’s private key
$V_{q}$	The data query vehicle
$\left(ID_{q},PID_{q}\right)$	$V_{q}$’s real identity and pseudo identity
$\left(x_{q},R_{q}\right)$	$V_{q}$’s private key
$\left(u_{k},v_{k}\right)$	Identifier of the segment *k*
*d*	Maximum value of sensory data
*m*	The total number of segments
*n*	The total number of fog nodes
δ	The total number of vehicles
|d|	Maximum length of sensory data
φ	The vehicles’ sharing key
$d_{i,k}^{j}$	The sensory data captured by $V_{i}$ at segment *k* under $FN_{j}$
$e_{i,k}^{j}$	If $d_{i,k}^{j}>0$, then $e_{i,k}^{j}=1$; If $d_{i,k}^{j}=0$, then $e_{i,k}^{j}=0$.
$H_{i}$	Eight one-way hash functions, $H_{i}:{\left\{0,1\right\}}^{*}\to Z_{q}^{*},i=1,2,\cdot \cdot \cdot ,7,H_{8}:{\left\{0,1\right\}}^{*}\to {\left\{0,1\right\}}^{|d|-1}$.
⊕	The exclusive OR operation
p,q	Two large prime numbers
$F_{p}$	The finite field over *p*
G	An additive group with the order *q* on the elliptic curve *E* over $F_{p}$
*P*	A generator of G

**Table 2:** Security comparisons. Efficient privacy-preserving data sharing (EP${}^{2}$DS), *√* represents “satisfy” and × denotes “does not satisfy”.

Security	S1	S2	S3	S4	S5	S6	S7	S8	S9	S10
Rabieh et al.’s scheme [17]	*√*	*√*	×	×	×	*√*	*√*	*√*	*√*	*√*
Sun et al.’s scheme [19]	*√*	*√*	×	*√*	*√*	*√*	*√*	*√*	*√*	*√*
Kong et al.’s scheme [23]	*√*	*√*	*√*	×	×	*√*	*√*	×	*√*	×
Paulet et al.’s scheme [25]	×	*√*	*√*	×	×	*√*	×	×	×	×
Zhu et al.’s scheme [26]	*√*	*√*	*√*	×	×	*√*	*√*	×	*√*	×
EP${}^{2}$DS	*√*	*√*	*√*	*√*	*√*	*√*	*√*	*√*	*√*	*√*

**Table 3:** Runtime of cryptographic operation (millisecond).

Notations	Descriptions	Runtime
$T_{sm}$	Scalar multiplication operation in G	0.3851
$T_{\log}$	Solving the DL operation mod *p*	0.6438
$T_{e}$	The exponentiation operation in $G_{1}$	2.0289
$T_{m}$	The multiplication operation in $G_{1}$	1.4293
$T_{h}$	Map to point hash function operation	3.5819
$T_{p}$	Bilinear pairing operation in $G_{1}$	10.3092

**Table 4:** Comparison of computation costs.

Scheme	Data Collection Phase			Data Query Phase	
	$V_{i}$	FN	CC	$V_{a}$	FN
[17]	$2T_{m}$+$2T_{e}$	$T_{m}$+$T_{e}$+(w+$1)T_{p}$	$T_{e}$+(n+$1)T_{p}$	−	−
	= 6.9164 ms	= 10.3092*w*+13.7674 ms	=10.3092*n*+2.0289 ms		
[19]	$2T_{m}$+$T_{e}$+$T_{h}$	(w+$3)T_{m}$+$4T_{p}$	$T_{m}$+$nT_{e}$+$2T_{p}$	−	−
	= 15.1967 ms	= 1.4293*w*+45.5247 ms	=2.0289*n*+11.7385 ms		
[23]	$4T_{m}$+$4T_{e}$	$2wT_{m}$	$6nT_{m}$+$4nT_{e}$	$10T_{m}$+$7T_{e}$	$9T_{m}$+$7T_{e}$
	= 13.8328 ms	= 2.8586*w* ms	=16.6914*n* ms	=28.4953 ms	=27.0660 ms
[25]	−	−	−	$5T_{m}$+$9T_{e}$	6m$T_{m}$+(8m+ )Te
				=25.4066 ms	=24.8070*m*+6.0867 ms
[26]	−	−	−	$2T_{p}$+$5T_{e}$	$4T_{p}$+$4T_{m}$
				=30.7629 ms	=46.9540 ms
EP${}^{2}$DS	$5T_{sm}$	(w+$3)T_{sm}$	(n+$3)T_{sm}$+$2T_{\log}$	$11T_{sm}$+$2T_{\log}$	$8T_{sm}$
	=1.9255 ms	=0.3851*w*+1.1553 ms	=0.3851*n*+2.4429 ms	=5.5237 ms	=3.0808 ms

**Table 5:** Comparison of the communication costs.

Scheme	Data Collection Phase	Data Query Phase	
	Data Report Size	Query Report Size	Response Report Size
Rabieh et al.’s scheme [17]	260 bytes	−	−
Sun et al.’s scheme [19]	516 bytes	−	−
Kong et al.’s scheme [23]	1152 bytes	1152 bytes	1664 bytes
Paulet et al.’s scheme [25]	−	256 bytes	256*m*+128 bytes
Zhu et al.’s scheme [26]	−	324 bytes	320 bytes
EP${}^{2}$DS	172 bytes	172 bytes	148 bytes

## Appendix A

**Security Model**

The proposed EP${}^{2}$DS scheme should satisfy the confidentiality and unforgeability. The security is defined by the following two interaction games executed by a challenger C and an attacker A. A could make the following queries.

- **Hash queries**: Upon receiving the query, C returns a random value to A.
- **Extract queries**: Upon receiving the query on the pseudo identity $PID_{i}$, C returns a private key to A.
- **Signcryption queries**: Upon receiving the query on the message $m_{i}$ under $PID_{i}$, C returns a ciphertext to A.

**Definition** **A1** (Confidentiality)**.** *The proposed scheme is secure against indistinguishability under the chosen plaintext attack (IND-CPA), if any probabilistic polynomial-time attacker does not have the ability to win the below game with a non-negligible advantage.*

The IND-CPA is defined by the following game.

**Setup**: C generates the system parameters and returns to A.

**Phase 1**: A adaptively makes the hash, extract, and signcryption queries with polynomial bounded times.

**Challenge**: A chooses a challenging identity $PID_{i}^{*}$, picks two messages $m_{0}^{*}$ and $m_{1}^{*}$ and sends to C. C randomly picks b∈{0,1} and produces the ciphertext of message $m_{b}^{*}$ under $PID_{i}^{*}$. Finally, C returns the ciphertext to A.

**Phase 2**: A is able to adaptively perform the query in Phase 1 apart from that, it cannot make extract queries on $PID_{i}^{*}$.

**Guess**: A produces a guess $b^{\prime }\in \left\{0,1\right\}$. The advantage that A wins the game is

$$Adv_{A}^{IND-CPA}=|\mathrm{Pr}\left[b^{\prime }=b\right]-\frac{1}{2}|.$$

**Definition** **A2** (Unforgeability)**.** *The proposed scheme can achieve existential unforgeability against adaptive chosen message attacks (EUF-CMA), if any probabilistic polynomial-time attacker does not have the ability to win the below game with a non-negligible advantage.*

The EUF-CMA is defined by the following game.

**Initialization**: A selects a challenging pseudo identity $PID_{i}^{*}$ and transmits to C.

**Setup**: C generates the system parameters and returns to A.

**Queries**: A adaptively makes hash, extract and signcryption queries.

**Forgery**: A outputs a ciphertext on $m_{i}^{*}$ under $PID_{i}^{*}$, such that

- The ciphertext on $m_{i}^{*}$ under $PID_{i}^{*}$ is valid.
- $PID_{i}^{*}$ has not been requested in the extract queries.

## Appendix B

**Security Proof**

**Theorem** **A1.**
*The proposed EP${}^{2}$DS scheme can provide confidentiality if ElGamal encryption is secure against the IND-CPA.*

Supposing there is an attacker A is able to win the game defined in Definition 1 with a non-negligible probability ε, we can construct an algorithm B that could break the *IND-CPA* of ElGamal encryption with probability $\varepsilon^{\prime }$.

**Initialization**: The simulator S for ElGamal encryption generates the $\left\{p,q,P,G,P_{pub}\right)$ and transmits to B.

**Setup**: B chooses hash functions $H_{i}$: i=1,2,···,8 and a super-increasing sequence $\vec{a}$. Finally, B returns {p,q,P,G, $P_{pub},P_{sp},H_{1},H_{2},H_{3},H_{4},H_{5},H_{6},H_{7},H_{8},\vec{a}\}$ to A.

To keep the rapidly response and consistency, B maintains the following list:

- **$L_{H_{2}}$**: It consists of tuples $\left(PID_{i},R_{i},P_{pub},h_{i}\right)$.
- **$L_{H_{4}}$**: It consists of tuples $(PID_{i},R_{i},C_{i,1},C_{i,2},L_{i},$$T_{i},\tau_{i})$.
- **$L_{V_{i}}$**: It consists of tuples $\left(PID_{i},x_{i},R_{i}\right)$.

**Phase 1**: A adaptively is able to adaptively perform the following polynomial bounded times queries.

**$H_{2}$ queries**: A performs a query on $\left(PID_{i},R_{i},P_{pub}\right)$, B executes as follows:

- If $L_{H_{2}}$ contains $\left(PID_{i},R_{i},P_{pub},h_{i}\right)$, B responds with the previous value $h_{i}=H_{2}\left(PID_{i},R_{i},P_{pub}\right)$ to A.
- If $L_{H_{2}}$ does not contain $\left(PID_{i},R_{i},P_{pub},h_{i}\right)$, B randomly chooses a number $h_{i}\in Z_{q}^{*}$, adds $(PID_{i},R_{i},$ $P_{pub},h_{i})$ into $L_{H_{2}}$ and returns $h_{i}$ to A.

**$H_{4}$ queries**: A performs a query on $(PID_{i},R_{i},C_{i,1},C_{i,2},$ $L_{i},T_{i})$, B executes as follows:

- If $L_{H_{4}}$ contains $\left(PID_{i},R_{i},C_{i,1},C_{i,2},L_{i},T_{i},\tau_{i}\right)$, B responds with the previous value $\tau_{i}=H_{4}(PID_{i},R_{i},C_{i,1},$ $C_{i,2},L_{i},T_{i})$ to A.
- If $L_{H_{4}}$ does not contain $(PID_{i},R_{i},C_{i,1},C_{i,2},L_{i},$ $T_{i},\tau_{i})$, B randomly chooses a number $\tau_{i}\in Z_{q}^{*}$, adds $\left(PID_{i},R_{i},C_{i,1},C_{i,2},L_{i},T_{i},\tau_{i}\right)$ into $L_{H_{4}}$ and returns $\tau_{i}$ to A.

**Extract queries**: A performs a query on $PID_{i}$, B executes as follows:

- If $PID_{i}=PID_{i}^{*}$, B aborts the game.
- If $PID_{i}\neq PID_{i}^{*}$, B executes:
  - If $L_{V_{i}}$ contains $\left(PID_{i},x_{i},R_{i}\right)$, B returns $\left(x_{i},R_{i}\right)$ to A.
  - If $L_{V_{i}}$ does not contain $\left(PID_{i},x_{i},R_{i}\right)$, B randomly chooses $x_{i},h_{i}\in Z_{q}^{*}$ and makes $R_{i}=x_{i}P-h_{i}P_{pub}$. If $h_{i}$ already appear in $L_{H_{2}}$, B chooses another $x_{i}\in Z_{q}^{*}$ and tries again. B inserts $\left(PID_{i},x_{i},R_{i}\right)$ and $\left(PID_{i},R_{i},P_{pub},h_{i}\right)$ into $L_{V_{i}}$ and $L_{H_{2}}$, respectively. Finally, B returns the $\left(x_{i},R_{i}\right)$ to A.

**Signcryption queries**: A makes a query on the message $m_{i}$ under $PID_{i}$, B returns $m_{i}$ to S. S randomly chooses $t_{i}\in Z_{q}^{*}$ and computes $C_{i,1}=t_{i}P$, $C_{i,2}=t_{i}P_{cc}+m_{i}P$,and returns them to B. B produces a ciphertext $\{PID_{i},$ $R_{i},C_{i,1},C_{i,2},L_{i},\sigma_{i},T_{i}\}$ in accordance with the proposed scheme. Finally, B returns the ciphertext to A.

**Challenge**: A selects a challenging identity $PID_{i}^{*}$, picks two same length message $m_{0}^{*}$ and $m_{1}^{*}$ and sends them to B. Then B transmits them to S. S randomly chooses b∈{0,1}, $t_{i}^{*}\in Z_{q}^{*}$ and computes $C_{i,1}^{*}=t_{i}^{*}P$, $C_{i,2}^{*}=t_{i}^{*}P_{cc}+m_{b}^{*}P$, and returns them to B. B produce a ciphertext $\left\{PID_{i}^{*},R_{i}^{*},C_{i,1}^{*},C_{i,2}^{*},L_{i}^{*},\sigma_{i}^{*},T_{i}^{*}\right\}$ in accordance with the proposed scheme. Finally, B returns the ciphertext to A.

**Phase 2**: A is able to adaptively perform the query in Phase 1 apart from it cannot make a extract queries on $PID_{i}^{*}$.

**Guess**: B can output $b^{\prime }$ as its guess against the *IND-CPA* of ElGamal encryption.

**Probability analysis**: Supposing that A is able to make at most $q_{H_{2}}$ times $H_{2}$ queries, $q_{H_{4}}$ times $H_{4}$ queries, $q_{e}$ times extract queries and $q_{s}$ times signcryption queries. We define two events as follows:

- $E_{1}$: B does not abort above game in extract queries.
- $E_{2}$: B is able to correctly output the value of *b*.

According to the above simulation, we could obtain that $\mathrm{Pr}\left[E_{1}\right]\geq {\left(1-\frac{1}{q_{H_{2}}}\right)}^{q_{e}}$ and $\mathrm{Pr}[E_{2}|E_{1}]\geq \varepsilon$, and hence the advantage that B is able to break the *IND-CPA* of ElGamal encryption is

$$\varepsilon^{\prime }=\mathrm{Pr}\left[E_{2}|E_{1}\right]\mathrm{Pr}\left[E_{1}\right]\geq {\left(1-\frac{1}{q_{H_{2}}}\right)}^{q_{e}}\varepsilon .$$

In accordance with the above analysis, we can conclude that B can break the *IND-CPA* of ElGamal encryption with a non-negligible probability, this is contradicts with the security of ElGamal encryption, so the proposed EP${}^{2}$DS scheme could provide confidentiality.

**Theorem** **A2.**
*The proposed EP${}^{2}$DS scheme can provide the unforgeability if the ECDL problem is hard.*

Assuming that there is an attacker A can break the unforgeability of the proposed EP${}^{2}$DS scheme with a non-negligible advantage ε, we can construct an algorithm B for solving the ECDL problem with probability $\varepsilon^{\prime }$.

**Initialization**: A picks a challenging identity $PID_{i}^{*}$ and returns to B.

**Setup**: Given an instance (P,aP=Q) of the ECDL problem, then B sets $P_{pub}=Q$ and returns $\{p,q,P,G,P_{pub},P_{sp},H_{1},$ $H_{2},H_{3},H_{4},H_{5},H_{6},H_{7},H_{8},\vec{a}\}$ to A.

**$H_{2}$ queries**: It is the same as Theorem 1.

**$H_{4}$ queries**: It is the same as Theorem 1.

**Extract queries**: It is the same as Theorem 1.

**Signcryption queries**: A makes a query on the message $m_{i}$ under $PID_{i}$, B executes as follows:

- If $PID_{i}=PID_{i}^{*}$, B randomly selects $t_{i},l_{i},\sigma_{i},h_{i},\tau_{i}\in Z_{q}^{*}$ and calculates $C_{i,1}=t_{i}P$, $C_{i,2}=t_{i}P_{cc}+m_{i}P$, $L_{i}=l_{i}P,R_{i}=\sigma_{i}P-\left(h_{i}P_{pub}+\tau_{i}L_{i}\right)$. If the $h_{i}$ already appears in $L_{H_{2}}$ or $\tau_{i}$ already appears in $L_{H_{4}}$, B chooses another $\sigma_{i}\in Z_{q}^{*}$ and tries again. Then, B returns the ciphertext $\{PID_{i},R_{i},C_{i,1},C_{i,2},L_{i},\sigma_{i},$ $T_{i}\}$ to A, and inserts $\left(PID_{i},R_{i},P_{pub},h_{i}\right)$ and $(PID_{i},R_{i},C_{i,1},C_{i,2},$ $L_{i},T_{i},\tau_{i})$ into $L_{H_{2}}$ and $L_{H_{4}}$, respectively.
- If $PID_{i}\neq PID_{i}^{*}$, B generates a ciphertext $\{PID_{i},R_{i},$ $C_{i,1},C_{i,2},L_{i},\sigma_{i},T_{i}\}$ in accordance with the proposed scheme. Then, B returns the ciphertext to A.

**Forgery**: A outputs a forged ciphertexts $\{PID_{i}^{*},R_{i}^{*},$ $C_{i,1}^{*},C_{i,2}^{*},L_{i}^{*},\sigma_{i}^{*},T_{i}^{*}\}$ on $m_{i}^{*}$ under $PID_{i}^{*}$. On the basis of the forking lemma [40,41], B is able to output another valid ciphertext $\left\{PID_{i}^{*},R_{i}^{*},C_{i,1}^{*},C_{i,2}^{*},L_{i}^{*},\sigma_{i}^{{*}^{\prime }},T_{i}^{*}\right\}$ on $m_{i}^{*}$ under $PID_{i}^{*}$ by choosing a different $H_{2}$. Since both ciphertexts are valid, we are able to gain the following two equations

$$\sigma_{i}^{*}P=R_{i}^{*}+h_{i}^{*}P_{pub}+\tau_{i}^{*}L_{i},\sigma_{i}^{{*}^{\prime }}P=R_{i}^{*}+h_{i}^{{*}^{\prime }}P_{pub}+\tau_{i}^{*}L_{i}.$$

We can gain the equations:

$$\begin{array}{c} \left(\sigma_{i}^{*}-\sigma {}_{i}^{{*}^{\prime }}\right)P=\sigma_{i}^{*}P-\sigma {}_{i}^{{*}^{\prime }}P=\left(h_{i}^{*}-h{}_{i}^{{*}^{\prime }}\right)P_{pub}=\left(h_{i}^{*}-h{}_{i}^{{*}^{\prime }}\right)aP. \end{array}$$

B outputs $a={\left(h_{i}^{*}-h_{i}^{{*}^{\prime }}\right)}^{-1}\left(\sigma_{i}^{*}-\sigma_{i}^{{*}^{\prime }}\right)$ as a solution of ECDL problem.

**Probability analysis**: Supposing that A is able to make at most $q_{H_{2}}$ times $H_{2}$ queries, $q_{H_{4}}$ times $H_{4}$ queries, $q_{e}$ times extract queries, and $q_{s}$ times signcryption queries. We define three events as follows:

- $E_{1}$: B never abort above game in extract and signcryption queries.
- $E_{2}$: B is able to output a valid ciphertext.
- $E_{3}$: $PID_{i}=PID_{i}^{*}$.

According to the above simulation, we could obtain that $\mathrm{Pr}\left[E_{1}\right]\geq {\left(1-\frac{1}{q_{H_{2}}}\right)}^{q_{e}}{\left(1-\frac{1}{q_{H_{4}}}\right)}^{q_{s}}$, $\mathrm{Pr}[E_{2}|E_{1}]\geq \varepsilon$, and $\mathrm{Pr}\left[E_{3}|E_{1}\wedge E_{2}\right]\geq \frac{1}{q_{H_{2}}}$. Thus, the probability that B is able to solve the ECDL problem is shown as:

$$\begin{array}{c} \varepsilon^{\prime }=\mathrm{Pr}\left[E_{1}\wedge E_{2}\wedge E_{3}\right]\geq \mathrm{Pr}\left[E_{3}|E_{1}\wedge E_{2}\right]\mathrm{Pr}\left[E_{2}|E_{1}\right]\mathrm{Pr}\left[E_{1}\right]\geq \frac{1}{q_{H_{2}}}{\left(1-\frac{1}{q_{H_{2}}}\right)}^{q_{e}}{\left(1-\frac{1}{q_{H_{4}}}\right)}^{q_{s}}\varepsilon . \end{array}$$

Due to the non-negligibility of ε, we are able to know that $\varepsilon^{\prime }$ is non-negligible. In accordance with the above analysis, we are able to conclude that B can solve the ECDL problem with a non-negligible probability. This contradicts with the hardness of the ECDL problem [42], and hence the proposed EP${}^{2}$DS scheme can provide unforgeability.
